# Epigenetic changes of mesenchymal stem cells in three-dimensional (3D) spheroids

**DOI:** 10.1111/jcmm.12336

**Published:** 2014-08-05

**Authors:** Ling Guo, Ying Zhou, Shan Wang, Yaojiong Wu

**Affiliations:** a The Shenzhen Key Laboratory of Health Sciences and Technology, Graduate School at Shenzhen, Tsinghua UniversityShenzhen, China; b School of Life Sciences, Tsinghua UniversityBeijing, China

**Keywords:** mesenchymal stem cells, miRNA, histone acetylation, 3D culture, multi-potency

## Abstract

Mesenchymal stem cells (MSCs) hold profound promise in tissue repair/regeneration. However, MSCs undergo remarkable spontaneous differentiation and aging during monolayer culture expansion. In this study, we found that 2–3 days of three-dimensional (3D) spheroid culture of human MSCs (hMSCs) that had been expanded in monolayer for six passages increased their clonogenicity and differentiation potency to neuronal cells. Moreover, in accordance with these changes, the expression levels of miRNA which were involved in stem cell potency were changed and levels of histone H3 acetylation in K9 in promoter regions of Oct4, Sox2 and Nanog were elevated. Our results indicate that spheroid culture increases their multi-potency and changes the epigenetic status of pluripotent genes in hMSCs.

## Introduction

Mesenchymal stem cells (MSCs) are self-renewing and expandable stem cells 1,2. They are capable of differentiating into mesoderm-and non-mesoderm-derived tissues 1,3. Residing in various tissues, MSCs are likely to participate in the maintenance of stem cell niches and tissue homoeostasis 4,5. Increasing evidence has suggested profound therapeutic potential of MSCs for a variety of diseases such as myocardial infarction, neural diseases, strokes and wounds 3. Moreover, MSCs show low immunogenicity and have immune regulatory properties 6. For these reasons, MSCs are emerging as an extremely promising therapeutic agent and numerous clinical trials for a variety of diseases are underway 7,8.

Mesenchymal stem cells represent as a rare cell population in the bone marrow (BM) and other tissues. Therefore, culture expansion of MSCs is an indispensable procedure to obtain sufficient amounts of cells for clinical therapies and tissue engineering. MSCs are commonly cultured as two dimensional (2D) monolayers. These methods could provide adequate cells for clinical therapies. However, MSCs age rapidly in these cultures and undergo considerable property changes, though they are considered genetically stable upon limited expansion 9. There are three main transcription factors, Oct4, Sox2 and Nanog, which govern embryonic stem cell self-renewal and pluripotency 10,11. These factors are also expressed in MSCs and appear to regulate their multi-potency 12. Telomerase reverse transcriptase (TERT) enables cells to divide repeatedly and reduced expression of the gene is associated with cell aging 13. Associated with the aging of MSCs in culture, rapid down-regulated expressions of these genes have been found 14. This has raised concerns over the therapeutic efficacy and safety of MSCs 15. Hence, the improvement of culture conditions to retain the primitive feature and therapeutic potential of MSCs has become a focus of research. Several recent studies showed that MSCs cultured in three-dimensional (3D) spheroids exhibited enhanced anti-inflammatory effect 16 and secreted higher levels of several cytokines such as VEGF, basic fibroblast growth factor (b-FGF), angiogenin, procathepsin B, interleukin (IL)-11 and bone morphogenic protein 2 17. Moreover, spheroid MSCs appear to exhibit increased therapeutic potential for myocardial ischaemia 18. However, the molecular mechanisms underlying phenotypical changes of MSCs in 3D spheroid culture condition have not been fully understood.

In this study, we found that 2–3 days of 3D spheroid culture of human MSCs (hMSCs) that had been expanded in 2D monolayer for six passages increased their expression levels of ‘stemness’ genes such as Oct4, Sox2 and Nanog and colony-forming activity. In accordance with these changes, hMSCs in 3D spheroids underwent the epigenetic alterations of ‘stemness’ genes including the expression levels of miRNAs associated with stem cell potency and elevated levels of histone H3 acetylation in K9 in promoter regions of Oct4, Sox2, Nanog and TERT.

## Materials and methods

### Cell isolation and culture

Human MSCs were isolated from human placenta as described previously 19. Briefly, term (38–40 weeks' gestation) placentas from healthy donors were harvested with written informed consent and the procedure was approved by the Ethics Committee of Xili Hospital. The placental tissue was washed several times with cold PBS and then mechanically minced and enzymatically digested with 0.25% trypsin-EDTA for 30 min. at 37°C in a water bath. The digest was subsequently filtered, pelleted and re-suspended in a growth medium consisting of DMEM (Gibco-Invitrogen, Carlsbad, CA, USA), 10% faetal bovine serum (FBS; Gibco-Invitrogen) and antibiotics. Cells were seeded on uncoated polystyrene dishes and medium was replaced every 2 days to reach 80% confluence. Cells were subcultured after trypsinization. To form spheroids, passage 5–7 hMSCs were cultured by a hanging drop method as described previously 16 with modifications. Briefly, hMSCs were plated in hanging drops in 35 μl DMEM containing 0–30% FBS and 300–300,000 cells per drop and incubated for up to 100 hrs (3000 hMSCs per drop in 10% FBS and 36 hrs of incubation were finally selected for subsequent experiments). Then the spheroids were transferred to a suspension culture and incubated in fresh growth medium for 24 hrs. To obtain single cells from spheroids, spheroids were incubated with 0.25% trypsin/EDTA for 6–10 min. (depending on the size of spheroids) with gentle pipetting every 2–3 min.

### Cell proliferation

MTT [3-(4, 5-dimethylthiazol-2-yl)-2,5-diphenyltetrazolium bromide] assay was used to compare the proliferation of hMSCs cultured in regular monolayer condition or in 3D spheroid culture. 10,000 hMSCs per well in 200 μl growth medium were seeded onto 96-well plates and incubated for 16 hrs. MTT was added to the culture medium to yield a final MTT concentration of 0.5 mg/ml and incubated in a CO_2_ incubator for 4 hrs. The cells were then collected and dissolved in dimethyl sulfoxide and subjected to colorimetric analysis at 570 nm.

### Flow cytometry

Single cell suspensions of hMSCs cultured in regular monolayer condition or in 3D spheroids were obtained by trypsinization. For apoptosis assay, freshly harvested cells were stained with an Annexin V-FITC apoptosis detection kit (Invitrogen) according to the supplier's instructions and analysed by flow cytometry.

### CFU-F assay

Colony-forming unit-fibroblast (CFU-F) assay was performed as previously described 16 with minor modifications. Monolayer or spheroid-derived hMSCs at passage six were seeded in 6-well culture plate at a density of 200 cells per well in the growth medium which was changed every 3 days. After 14 days of culture, the cells were fixed with 4% paraformaldehyde (PFA, Sigma-Aldrich) and stained with 0.1% crystal violet solution (Sigma-Aldrich). The number of colonies (diameter ≥2 mm) was counted.

### Differentiation of hMSCs

Chemicals used in differentiation assays were purchased from Sigma-Aldrich except for indication. hMSCs derived from monolayers or spheroids were dissociated and seeded in tissue culture plates for differentiation analyses. Adipogenic differentiation was induced in DMEM supplemented with 10% FBS, 1% penicillin-streptomycin, 500 μM 3-isobutyl-1-methylxanthine (IBMX), 1 μM dexamethasone, 10 μM insulin and 200 μM indomethacin. After 2 weeks, cells were fixed in 4% PFA and stained with Oil Red O to observe lipid droplets 3,19.

Osteogenic differentiation was induced by culturing hMSCs in DMEM-high glucose (DMEM-HG) supplemented with 10% FBS, 1% penicillin-streptomycin, 10 nM dexamethasone, 50 μM ascorbic acid 2-phosphate and 10 mM β-glycerophosphate. After 2 weeks, cells were fixed in 4% PFA and stained with Alizarin red S to observe mineralized matrix deposition 3,19.

To induce neuron differentiation, monolayer cells and spheroids were dispersed into single cells and seeded on tissue culture plates. After incubation in the growth medium overnight, the medium was changed to induction medium consisting of DMEM-HG supplemented with 1% FBS, 1% penicillin-streptomycin and 100 ng/ml bFGF and incubated for 24 hrs, then 20 ng/ml epidermal growth factor (Roche, Basel, Switzerland), 20 ng/ml bFGF, 25 ng/ml nerve growth factor, 0.5 mM IBMX and 1 mM dbcAMP were added to the culture and incubated for another 7 days 20,21. Total RNA was extracted to determine the expression of βIII-tubulin and neurofilament-L (NF-L) real-time polymerase chain reaction (real-time PCR) analysis. Primer sets are listed in Table1.

**Table 1:** Real-time PCR primers

		Oligonucleotide (5′–3′)
Genes (for mRNA)		
Oct4	Forward Reverse	5′-GTATTCAGCCAAACGACCATC-3′ 5′-CTGGTTCGCTTTCTCTTTCG-3′
Nanog	Forward Reverse	5′-AATACCTCAGCCTCCAGCAGATG-3′ 5′-TGCGTCACACCATTGCTATTCTTC-3′
Sox2	Forward Reverse	5′-GGGAAATGGGAGGGGTGCAAAAGAGG-3′ 5′-TTGCGTGAGTGTGGATGGGATTGGTG-3′
TERT	Forward Reverse	5′-CGGAAGAGTGTCTGGAGCAA-3′ 5′-GGATGAAGCGGAGTCTGGA-3′
ALP	Forward Reverse	5′-ACTGGTACTCAGACAACGAGAT-3′ 5′-ACGTCAATGTCCCTGATGTTATG-3′
OPN	Forward Reverse	5′-TTGCAGCCTTCTCAGCCAA-3′ 5′-GGAGGCAAAAGCAAATCACTG-3′
FABP4	Forward Reverse	5′-AAAGTCAAGAGCACCATAACC-3′ 5′-TTCAATGCGAACTTCAGTCC-3′
PPARr2	Forward Reverse	5′-GCGATTCCTTCACTGATAC-3′ 5′-TCAAAGGAGTGGGAGTGGTC-3′
βIII-tubulin	Forward Reverse	5′-ACAATTTCATCTTTGGTCAGAGTGG-3′ 5′-TCACACTCCTTCCGCACCAC-3′
NF-L	Forward Reverse	5′-ACAAGCAGAACGCCGACATC-3′ 5′-TCCAAAGCCATCTTCACGTTG-3′
GAPDH	Forward Reverse	5′-CGTGGAAGGACTCATGACCA-3′ 5′-TCCAGGGGTCTTACTCCTTG-3′
miRNAs		
Hsa-miR-489	Forward	5′-GTGACATCACATATACGGCAGC-3′
Hsa-miR-17-5p	Forward	5′-CAAAGTGCTTACAGTGCAGGTAG-3′
Hsa-miR-145-5p	Forward	5′-GTCCAGTTTTCCCAGGAATCCCT-3′
Hsa-miR-24-3p	Forward	5′-TGGCTCAGTTCAGCAGGAACAG-3′
Hsa-miR-370	Forward	5′-GCCTGCTGGGGTGGAACCTGGT-3′
Hsa-miR-7-5p	Forward	5′-TGGAAGACTAGTGATTTTGTTGT-3′
Hsa-miR-433	Forward	5′-ATCATGATGGGCTCCTCGGTGT-3′
Genes (for ChIP)		
GAPDH	Forward Reverse	5′-TACTAGCGGTTTTACGGGCG-3′ 5′-TCGAACAGGAGGAGCAGAGAGCGA-3′
Sox2	Forward Reverse	5′-AGTTGGACAGGGAGATGGC-3′ 5′-AACCTTCCTTGCTTCCACG-3′
Oct4	Forward Reverse	5′-CTTCCACAGACACCATTGCC-3′ 5′-AGTCCCACCCACTAGCCTTG-3′
Nanog	Forward Reverse	5′-GCCCTATCCAAATCCTATCACTT-3′ 5′-GGTCAGCACAAAATACAGGTCA-3′
TERT	Forward Reverse	5′-GGCTCCCAGTGGATTCGC-3′ 5′-GGAGGCGGAGCTGGAAGG-3′
ALP	Forward Reverse	5′-TGTTGACAGACACAGAGACAGACG-3′ 5′-GTCGGCATCTTCCTTCTGCG-3′
OPN	Forward Reverse	5′-GAGACATATTTCCCCCTACC-3′ 5′-CAGTTGTGAAATGCAGATTGCAC-3′
FABP4	Forward Reverse	5′-GGATGGCCTTGGACTCACTC-3′ 5′-AGAAACACCACAGGAGGCTGA-3′
PPARr2	Forward Reverse	5′-TTAGCAGTTTGGCACAGCTAGG-3′ 5′-TCAGGAAAACTCTGGCTTCTTG-3′

### Real-time PCR analysis

Total RNA was extracted from hMSCs with TRIzol (Invitrogen) following the manufacturer's instructions. First-strand cDNA was prepared by reverse transcription with Superscript II reverse transcriptase (Invitrogen) and oligo(dT) primers and stored at −20°C. Real-Time PCR was performed with SYBR Green Real-Time PCR Master Mix (TOYOBO, Osaka, Japan) on an ABI 7300 QPCR System. As an internal control, levels of glyceraldehyde-3-phosphate dehydrogenase (GAPDH) were quantified in parallel with target genes. Normalization and fold changes were calculated using the ΔΔCt method. Primer sets are listed in Table1.

For analysis of miRNA expression, real-time PCR was carried out with All-in-One miRNA qRT-PCR Detection System kit (Genecopoeia, Guangzhou, China) under the following conditions: 37°C, 30 min.; 95°C, 10 min. of reverse transcription; 95°C, 3 min.; 95°C, 15 sec.; 60°C, 30 sec. for the amplification. 5S rRNA was used as an internal control. The primers of 5S rRNA and let-7f were purchased from Genecopoeia. The first primers are listed in Table1.

### Immunofluorescence

Human MSCs grown on coverslips were fixed in 1% PFA. Human MSC spheroids were fixed in 3% PFA, embedded in OCT and cryosectioned (6 μm thickness). After permeabilization with 0.1% Triton X-100, samples were incubated with a primary antibody against βIII-tubulin (Sigma-Aldrich) overnight at 4°C, followed by detection with a fluorescence-conjugated secondary antibody. Nuclei were stained with 4, 6-diamidino-2-phenylindole (DAPI). After mounting, samples were visualized under confocal microscope (FV1000; Olympus, Tokyo, Japan).

### Chromatin immunoprecipitation

A double chromatin immunoprecipitation (ChIP) assay was performed with passage six hMSCs, according to the fast ChIP method 22, except that the protein-A sepharose was replaced by protein G Dynabeads (Life Technologies, Carlsbad, CA, USA) and that the antibodies (against H3, cat#:2650s, and H3K9ac, cat#: 9649s; Cell Signaling Technology, Danvers, MA, USA) were incubated with samples overnight at 4°C. Normal rabbit IgG (Cell Signaling Technology) was used for mock IP. The purified DNA was measured by quantitative fluorescent PCR analysis using SYBR Green real-time PCR Master Mix (TOYOBO) on an ABI 7300 QPCR System. Primer sets were listed in Table1. Data were analysed using Per cent Input Method (Life Technologies), in which equal amounts of starting chromatin were used as input, and signals obtained from the ChIP over the background were divided by signals obtained from an input sample. The results were counted with the formula: per cent input = 100% × (2^Ct (input)−Ct (antibody)^ − 2^Ct (input)−Ct (mock)^). The above experiments were repeated at least three times and hMSCs derived from three donors were used.

### Western blotting

Total cell lysates were prepared by incubation of cells in RIPA lysis buffer containing protease inhibitors. 5 μg proteins were loaded per lane. Total protein was separated by 12% SDS-PAGE and blotted to polyvinylidene difluoride membranes. The membranes were blocked with 5% non-fat dried milk and incubated overnight at 4°C with a rabbit anti-Histone H3 antibody (cat#:2650s; Cell Signaling Technology) or with a rabbit anti-Acetyl-Histone H3(Lys9) antibody (cat#: 9649s; Cell Signaling Technology). The membranes were washed, then incubated with a horseradish peroxidase-conjugated goat anti-rabbit IgG antibody and finally visualized by chemiluminescence (ECL kit; Bio-Rad, Hercules, CA, USA). The blots were quantified using Image J software 23, and the areas of control were regarded as 1.

### Statistical analysis

All data were expressed as mean ± SD. Statistical Package for the Social Sciences (SPSS) 13.0 software package was used for Student's *t*-test and statistical significance was defined as *P* < 0.05.

## Results

### Formation of hMSC spheroids

**Figure 1:**
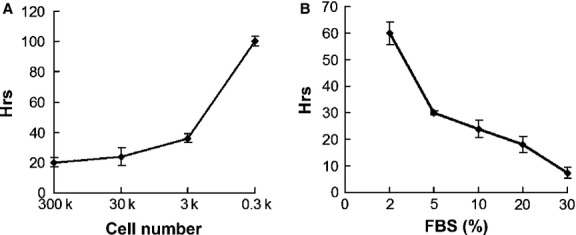
Influences of cell number and serum concentration on spheroid formation. 300 to 300,000 hMSCs (as indicated in **A**) per hanging drop of 35 μl DMEM containing 0–30% FBS (as indicated in **B**) were plated and incubated for up to 96 hrs to determine the time taken to form spheroids. In the absence of FBS, hMSCs did not form solid spheroids in hanging drops in 96 hrs. The experiment was repeated three times with similar results.

**Figure 2:**
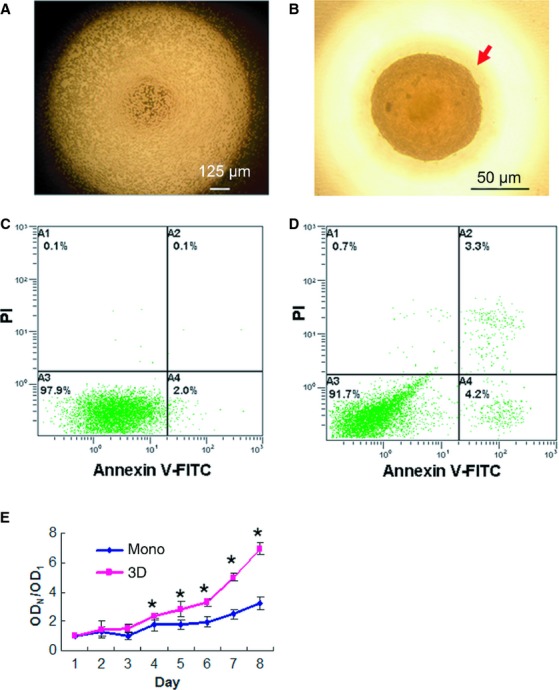
Viability and proliferation of spheroid hMSCs. 3 × 10^4^ hMSCs were suspended in 10% FBS in a hanging drop and incubated for 36 hrs to form spheroids. (**A**) It shows a microscopic image of the hanging drop at 0 hr. (**B**) A representative sphere of the hanging drop at 36 hrs was photographed under microscope. The spheroids were then transferred to suspension culture and incubated for 24 hrs. Cells derived from the spheroids were subjected to Annexin V/PI analysis by flow cytometry (**D**). hMSCs that were in the same passage but cultured in monolayer were used as a control (**C**). (**E**) MTT assay. Single hMSCs derived from spheroids (3D) or monolayer (mono) were seeded into tissue culture plates, incubated and subjected to MTT analysis. Spheroid hMSCs showed greater proliferating rates. Triplet wells were used for the assay, and the experiment was repeated three times with similar results (*P < 0.05). OD_N_ represents OD values in corresponding days, OD_N_/OD1 represents the ratio of OD_N_ to OD1 (the OD value in the first day).

To aggregate hMSCs, we first cultured hMSCs in hanging drops and investigated how the number of hMSCs and serum concentrations affected the formation of spheroids. As expected, increasing cell numbers and serum (FBS) concentrations were associated with decreasing time taken to form spheroids (Fig.1A and B). In the absence of FBS, some hMSCs formed loose aggregates, but did not form solid spheroids. When 3 × 10^4^ hMSCs were suspended in 10% FBS per hanging drop of 35 μl, cells were initially evenly distributed in the hemisphere drop (Fig.2A) and gradually formed solid spheroids at 36 hrs (data not shown). Longer time in hanging drops led to reduced cell viability. To avoid cell death and allow sufficient time to change the epigenetic status of the cells, the spheroids were then transferred to suspension culture and incubated for another 24 hrs. Homogeneous populations of spheres could be maintained in suspension without significant agglomeration (Fig.2B). Human MSCs in the spheroids formed in this condition (hanging drop plus suspension) were easily dissociated after trypsinization and more than 90% cells remained viable as determined by Annexin V stain (Fig.2D). Compared to the conventional longer hours of hanging drop culture, our modified method significantly reduced cell death (data not shown). We obtained similar results using hMSCs derived from three donors. So the modified condition was used for subsequent experiments. To further evaluate the proliferation potential of cells derived from the spheroids, MTT assay was performed. Human MSC spheroids were dissociated with trypsin and seeded in culture plates. Equal numbers of hMSCs derived from monolayer culture in the same passage were used as control. hMSCs derived from spheroids exhibited greater proliferating rates compared to hMSCs derived from monolayer culture after the first few days in culture (Fig.2E, *P* < 0.05).

### Clonogenicity of spheroid hMSCs

**Figure 3:**
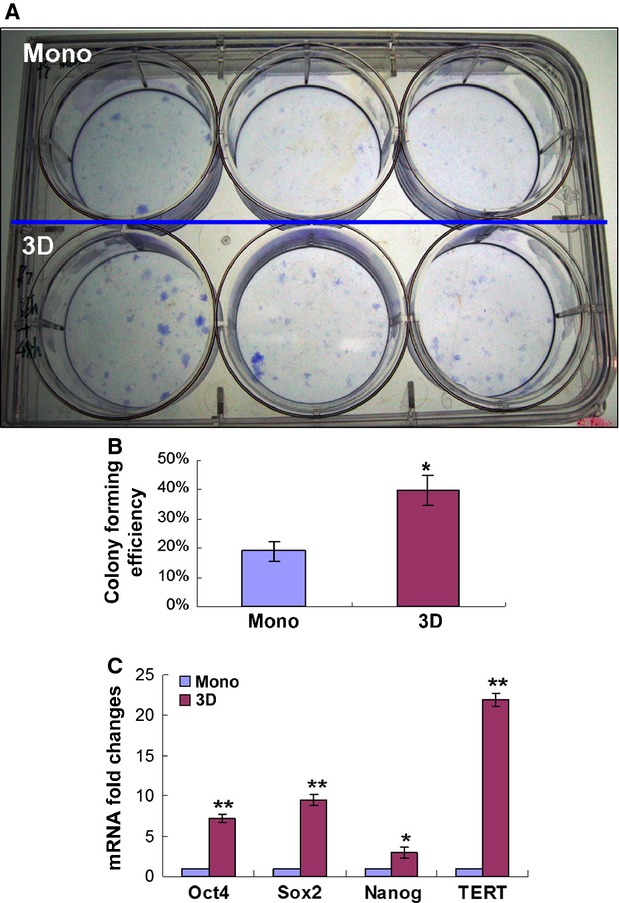
CFU-F assay and expression of pluripotent genes. Monolayer or spheroid (3D)-derived hMSCs were seeded in 6-well culture plate at a density of 200 cells per well and incubated for 14 days. Then the culture was fixed with 4% PFA and stained with crystal violet solution (**A**). The number of colonies (diameter ≥2 mm) was counted (**B**, *P < 0.05). Triplet wells were used for the assay, and the experiment was repeated three times with similar results. (**C**) Real-time PCR analysis of the expression of Oct4, Sox2, Nanog and TERT in monolayer or spheroid-derived hMSCs. The experiments were repeated four times with similar results. mRNA folds changes were relative to monolayer hMSCs. **P < 0.01, *P < 0.05.

To examine whether spheroid culture condition had altered the clonogenicity of hMSCs, we performed CFU-F assay. Consistent with a previous study 16, the dissociated spheroid hMSCs readily generated colonies when plated at clonal densities, and the number of colonies from spheroid cells was significantly more than that from the monolayer culture after 14 days of culture (Fig.3A and B, *P* < 0.05). Moreover, cells in colonies formed by spheroid hMSCs were more uniform and less flat compared to cells in colonies formed by monolayer hMSCs. In accordance with the above changes, spheroid hMSCs showed increased mRNA levels of Oct4, Sox2, Nanog and TERT genes, with 7.7-fold, 9.2-fold, 2.3-fold and 21.4-fold increases relative to monolayer hMSCs, respectively (Fig.3C).

### Multi-potent properties of spheroid hMSCs

**Figure 4:**
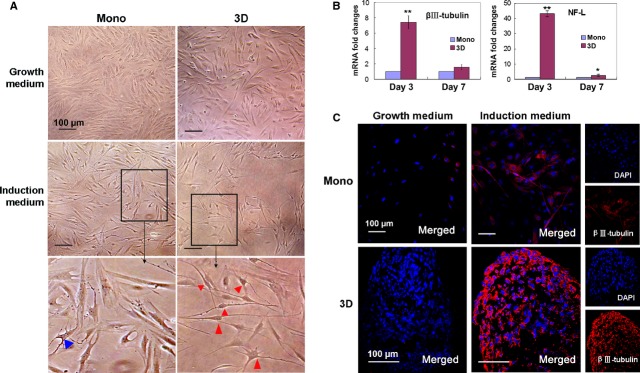
Neurogenic differentiation of hMSCs. (**A**–**C**) Morphological changes of hMSCs after neurogenic induction. Human MSCs derived from monolayer or spheroids (3D) were cultured in the growth medium or neurogenic induction medium for 3 days and photographed (**A**). Higher magnification images of the cells in the induction medium were shown in the bottom panel and cells with processes were indicated by arrow head. Triple wells were used for the experiment. The experiment was repeated three times with similar results and representative images from one experiment were shown. (**B** and **C**) Expression of neural genes in hMSCs before and after neurogenic induction. (**B**) Monolayer or spheroid hMSCs were cultured in the growth medium or neurogenic induction medium for 3 or 7 days and the expression levels of βIII-tubulin and NF-L genes were examined by real-time PCR analysis. The experiments were repeated three to four times with similar results. Folds changes were relative to monolayer hMSCs. **P < 0.01, *P < 0.05. (**C**) The cells cultured in the growth or neurogenic induction medium for 3 day were immunostained for the expression of βIII-tubulin (red) and photographed under confocal microscope. Nuclei were stained with DAPI (blue).

To examine whether spheroid culture increased the differentiation capacity of hMSCs, we cultured the cells in different induction media. Similar to a previous finding 16, spheroid hMSCs differentiated into osteoblasts (Fig. S1) and adipocytes (Fig. S2) when being cultured in osteogenic and adipo genic induction medium, respectively, and proportions of differentiated cells were similar to those in hMSCs derived from monolayer culture. We further examined the capability of neuronal differentiation. When cultured in neurogenic induction medium, a higher percentage of spheroid hMSCs developed processes compared to hMSCs derived from successive monolayer culture (Fig.4A). On day 3, the majority of hMSCs derived from spheroids exhibit a bipolar spindly phenotype, and multi-polar neuron-like cells were observed on day 7. In accordance with the morphological changes, real-time PCR analysis revealed that spheroid hMSCs expressed higher levels of neural specific genes βIII-tubulin and NF-L upon neurogenic induction for 3 and 7 days, compared to monolayer hMSCs (Fig.4B). Immunofluorescence analysis further confirmed the protein expression of βIII-tubulin in hMSCs spheroids (Fig.4C).

### miRNA expression in spheroid hMSCs

**Figure 5:**
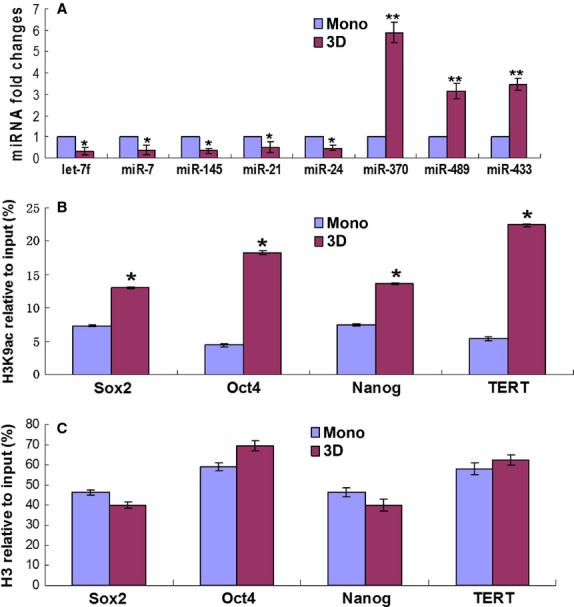
Alterations of miRNA expression and histone acetylation of ‘stemness’ genes in spheroid hMSCs. Monolayer or spheroid (3D) hMSCs were analysed for the expression of miRNAs potentially involved with pluripotency as indicated in (**A**) by real-time PCR analysis and alterations in histone H3 acetylation levels in lysine 9 (H3K9ac; **B**) and histone H3 (**C**) in the promoter regions of Oct4, Sox2, Nanog and TERT.

To understand whether miRNAs were involved with the phenotypical changes of hMSCs in spheroids, real-time PCR analysis was performed to examine the expression of miR-489, miR-370, miR-433, *let-7f*, miR-7, miR-145, miR-21 and miR-24. These miRNAs were selected for either of the two following reasons: they had been shown to participate in the regulation of pluripotency and differentiation of stem cells in previous studies 24–26, or they were predicted to target at the 3′ non-coding region of Sox-2, Oct-4 or Nanog using the PicTar database. Real-time PCR analysis showed that miR-489, miR-370 and miR-433 were highly expressed in spheroid hMSCs compared to monolayer hMSCs, especially miR-370 with a fivefold increase, while *let-7f*, miR-7, miR-145, miR-21 and miR-24 were down-regulated in spheroid hMSCs (Fig.5A). We used hMSCs derived from three donors and obtained similar results.

### Histone acetylation in spheroid hMSCs

**Figure 6:**
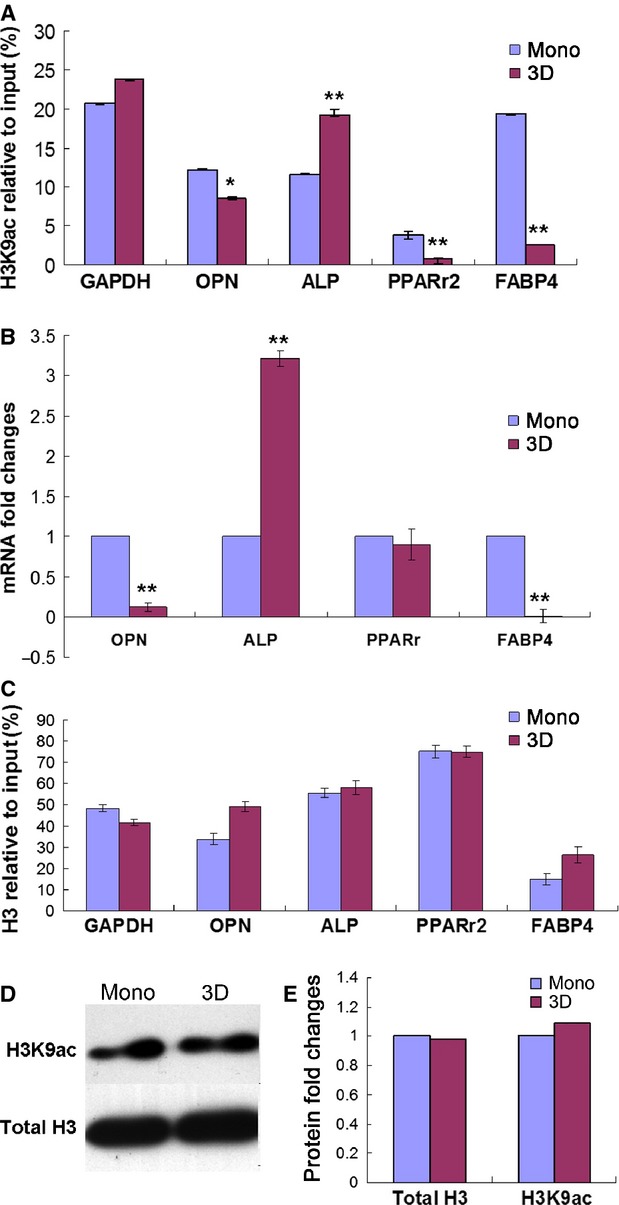
Histone acetylation alterations of OPN, ALP, PPARr2, FABP4 and GAPDH genes in spheroid hMSCs. (**A**) Levels of H3K9ac in the promoter regions of OPN, ALP, PPARr2, FABP4 and GAPDH in monolayer and 3D spheroid hMSCs as determined by ChIP assay. (**B**) Real-time PCR analysis of the mRNA expression levels of ALP, OPN, PPARr2 and FABP4. (**C**) Levels of histone H3 in the promoter regions of GAPDH, ALP, OPN, PPARr2 and FABP4 in monolayer and 3D spheroid hMSCs by ChIP assay. (**D** and **E**) Total histone H3 and H3K9ac levels in monolayer or 3D spheroid hMSCs were determined by Western blot (**D**) and the intensity of bands was quantified with Image J (**E**). The experiments were repeated two or three times with similar results. Fold changes were relative to monolayer hMSCs. *P < 0.05; **P < 0.01. OPN, osteopontin; ALP, alkaline phosphatase; PPARr2, peroxisome proliferator-activated receptors γ2; FABP4, fatty acid binding protein 4.

As described earlier, spheroid hMSCs exhibited higher mRNA levels of Oct4, Sox2, Nanog and TERT genes (Fig.3C). Associated with the gene expressional changes were increases in histone H3K9 acetylation levels in the promoter regions of these genes in spheroid hMSCs compared to monolayer hMSCs (Fig.5B), while histone H3 levels of these genes between spheroid hMSCs and monolayer hMSCs had no significant differences (*P* > 0.05, Fig.5C). Meanwhile, we also examined histone H3K9ac levels in housekeeping gene GAPDH and genes involved with osteogenic (osteopontin, OPN) and adipogenic (peroxisome proliferator-activated receptors γ2, PPARr2, and fatty acid binding protein 4, FABP4) in monolayer and 3D cultured hMSCs, and the results showed no differences in histone H3K9ac levels of GAPDH, but significant decreases in histone H3K9ac levels of OPN, PPARr2 and FABP4 (Fig.6A). Real-Time PCR analysis showed corresponding decreases in the expression of these genes except for PPARr2 (Fig.6B). Alkaline phosphatase (ALP) is a marker of stem cells and is also a gene involved in osteogenic differentiation. We found that the levels of histone H3K9ac and the expression of ALP were significantly increased after 3D culture (Fig.6A and B). However, there were no significant differences in histone H3 levels in the promoters of these genes (Fig.6C). To examine whether 3D spheroid culture caused global changes in H3K9ac, we evaluated total H3 and H3K9ac levels of hMSCs cultured in monolayer and 3D spheroids by Western blot, and the results showed no significant differences between these two groups (Fig.6D and E).

## Discussion

Accumulating evidence has indicated that MSCs undergo considerable changes in monolayer culture conditions, though they remain the basic features of MSCs such as surface expression of CD105, CD73 and CD90 and differentiation into osteoblasts, adipocytes and chondroblasts *ex vivo* 2. Culture expanded MSCs often exhibit signs of aging and spontaneous differentiation including increases in cell size and expression levels of osteogenic and adipogenic genes, and reduced capacities of trafficking and homing, production of paracrine factors, multi-potent differentiation and tissue repair 19,27,28. Such a loss of MSC potential is a major hurdle for the therapeutic use of these clinically relevant cells and it is thus important to develop techniques to preserve their primitive properties.

Previous studies suggest that 3D spheroid culture may improve the property of MSCs 16. It is tempting to examine whether spheroid culture condition could restore hMSCs to more primitive states. In this study, we cultured hMSCs in spheroids and examined the epigenetic status of pluripotent genes. To increase cell viability, we modified the hanging drop protocol 16 by shortening the hanging drop culture time but adding a suspension culture step. With this protocol, human MSCs that had been expanded in monolayer culture for several passages could be reversed to a ‘younger’ state after 2–3 days of spheroid culture while their viability was modestly affected. The cells exhibited increases in clonogenicity immediately following suspension culture. Meanwhile, the cells showed elevated expression levels of pluripotent genes such as Oct4, Nanog and Sox2 and TERT gene which enables repeated cell division after 3D spheroid culture. Moreover, the cells showed increased differentiation potential to neural cells besides retaining the capabilities of osteogenesis and adipogenesis. This may imply improved therapeutic potential of these cells for neurological diseases. In accordance with the expressional changes of these genes were epigenetic alterations in miRNAs and histone H3K9 acetylation levels.

Previous studies suggest that epigenetic dysregulations are main causes of phenotypical changes of MSCs in culture 19,29. Several recent studies indicate that the miRNAs are critically involved in stem cell pluripotency, proliferation and differentiation 24–26. In the present study, we examined the levels of a group of miRNAs, which had been reported to regulate the expression of pluripotent genes such as miR-489, miR-370 and miR-433 30, or were predicted to target stem cell pluripotency genes such as miR-7 and miR-21. Our results showed that miR-489, miR-370 and miR-433 were highly expressed in spheroid hMSCs, while miR-7, miR-145, let-7f, miR-21 and miR-24 were down-regulated in spheroid hMSCs, compared to hMSCs that had been cultured in monolayer. MiR-489, miR-370 and miR-433 have recently been found to play an important role in maintaining the quiescent state of adult stem cells 30. A quiescent status is necessary for cells to reverse to a status with increased plasticity. MiR-21, miR-24 and let-7f were reported to contribute to MSC differentiation 26, while miR-145 was found previously to inhibit hESC self-renewal, repress the expression of pluripotent genes and induce lineage-restricted differentiation by targeting the pluripotency factors Oct4, Sox2 and Klf4 24. Our results indicate that 3D culture environment has influenced the expression levels of miRNAs involved with MSC multi-potency and differentiation.

Acetylation of histone H3 in lysines has been found on euchromatin near genes that are actively being transcribed 29. Pluripotency factors such as Sox2, Oct4 and Nanog are main transcription factors that govern embryonic stem cells self-renewal and pluripotency 10,11. These factors are also expressed in MSCs of early passages 31,32. However, the expression levels of these factors decline markedly upon successive monolayer culture expansion 19,32. Meanwhile, the expression levels of osteogenic and adipogenic genes increase because of spontaneous differentiation 19,33. In this study, we found that spheroid culture increased the expression levels of pluripotent genes but decreased the expression levels of genes involved with osteogenic and adipogenic differentiation in hMSCs that had been cultured in monolayer for several passages. This is in agreement with a previous study where the expression of ‘stemness’ genes in MSCs increased after spheroid culture on chitosan or chitosan-HA (hyaluronan) membranes 34. Moreover, consistent with changes in gene expression, we found corresponding changes in H3K9ac level in the promoter regions of these genes. This is consistent with our previous findings on MSC aging *in vitro*, where histone H3 acetylation levels in K9 and K14, but not promoter DNA methlylation levels of TERT, Oct4, Sox2 and Nanog genes were closely co-related with expressional levels of these genes 19. Moreover, in a recent study, promoter DNA methlylation levels were found not to be associated with expression levels of Oct4 and Nanog in human umbilical cord and BM derived MSCs 31. These results suggest that histone modifications in promoters of certain genes are likely to play a critical role in regulating MSC self-renewal and differentiation. Previous studies show that no significant changes in global histone acetylation level in MSC aging 35,36. In agreement with these studies, we found that 3D spheroid culture did not alter the levels of total histone H3 and total H3K9ac. These results suggest that 3D spheroid culture is likely to selectively affect the histone H3 acetylation status of certain genes such as genes involved in multi-potency and differentiation of MSCs.

Spheroid MSCs have been shown to exhibit enhanced therapeutic potential in recent studies. They secreted higher levels of tumour necrosis factor-α stimulated protein 6 (TSG-6), an anti-inflammatory protein, and showed enhanced effects in repairing the infarcted myocardium after intravenous infusion in mice 16,37. Moreover, intramyocardial injection of MSC spheroids increased vascular density in the infarcted myocardium and improved cardiac function in rats 38. In the present study, we found that spheroid MSCs exhibited increased multi-potency, a potential mechanism to increase their therapeutic effect in tissue regeneration.

Taken together, 3D spheroid culture of *ex vivo* expanded hMSCs in the absence of exogenous substrates restored their stem cell properties, including enhanced differentiation potential into multi-lineage cells particularly into cells with neural features. Along with these changes were increased expression of pluripotent genes and decreased expression of genes involved in osteogenic and adipogenic differentiation. In accordance with expressional changes of these genes, evident epigenetic alterations of these genes were found including miRNA expression and histone H3K9 acetylation status. Our data suggest that 3D spheroid culture changes the epigenetic status of stemness genes in successive expanded MSCs, improve their multi-potency, and thus may serve as an effective approach for ameliorating MSC aging and enhancing their therapeutic potential.

## Supporting Information

Figure S1 Osteogenic differentiation of hMSCs. Human MSCs derived from monolayer and spheroids (3D) were cultured in the growth medium or osteogenic induction medium for 14 days. Cells were photographed after staining with Alizarin red for detection of osteogenesis. The experiment was repeated three times with similar results and results from one experiment were shown.

Figure S2 Adipogenic differentiation of hMSCs. Human MSCs derived from monolayer and spheroids (3D) were cultured in the growth medium or adipogenic induction medium for 14 days. Cells were photographed after staining with Oil Red O for detection of adipogenesis. The experiment was repeated three times with similar results and results from one experiment were shown.
