# Supplementary figures and images for: Epigenetic changes of mesenchymal stem cells in three-dimensional (3D) spheroids
Source: J Cell Mol Med. 2014 Aug 5;18(10):2009–19. doi: 10.1111/jcmm.12336 (PMC4244016; doi:10.1111/jcmm.12336)

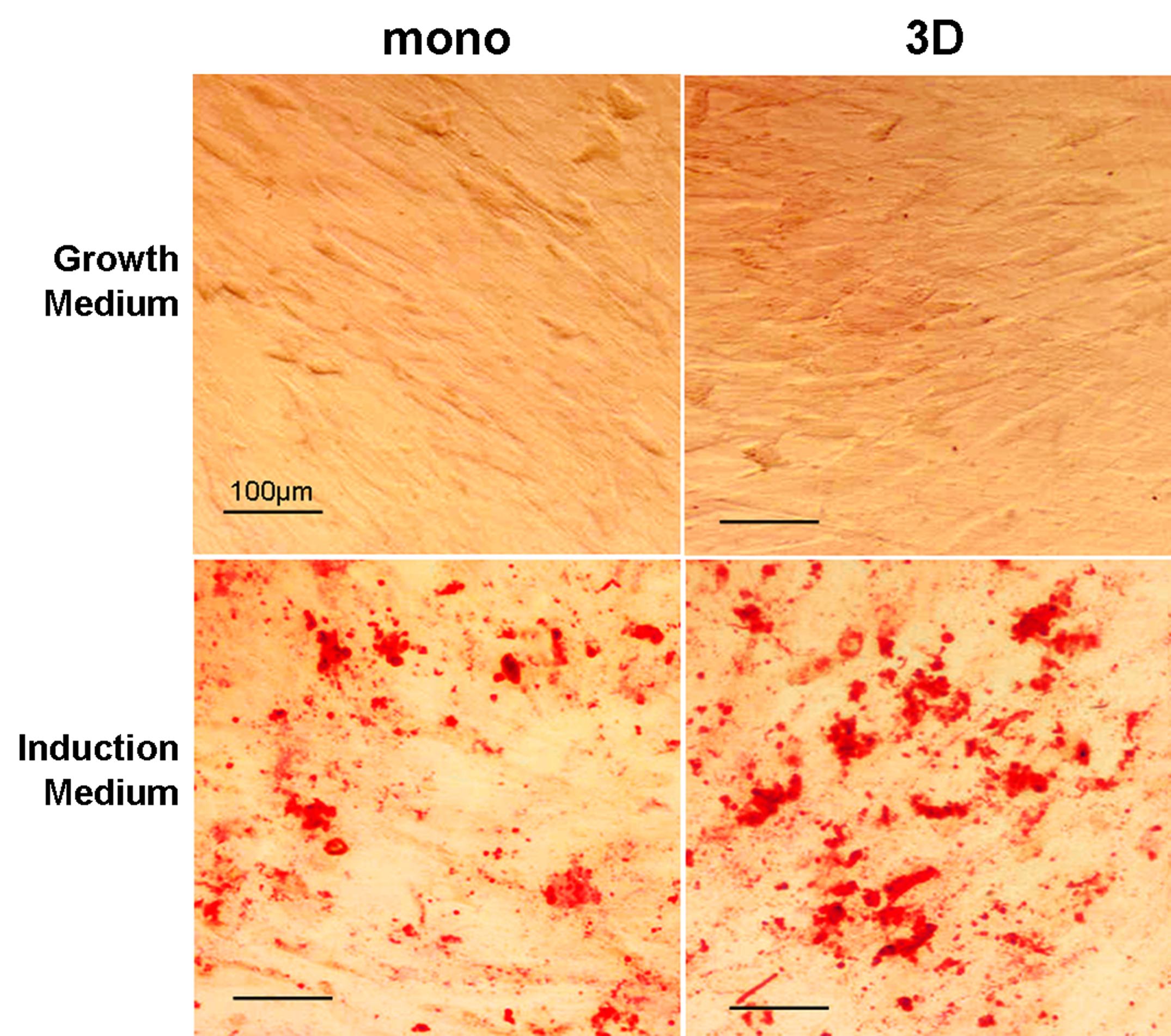

Supplement: Figure S1 — Osteogenic differentiation of hMSCs. Human MSCs derived from monolayer and spheroids (3D) were cultured in the growth medium or osteogenic induction medium for 14 days. Cells were photographed after staining with Alizarin red for detection of osteogenesis. The experiment was repeated three times with similar results and results from one experiment were shown. [file jcmm0018-2009-sd1.jpg]

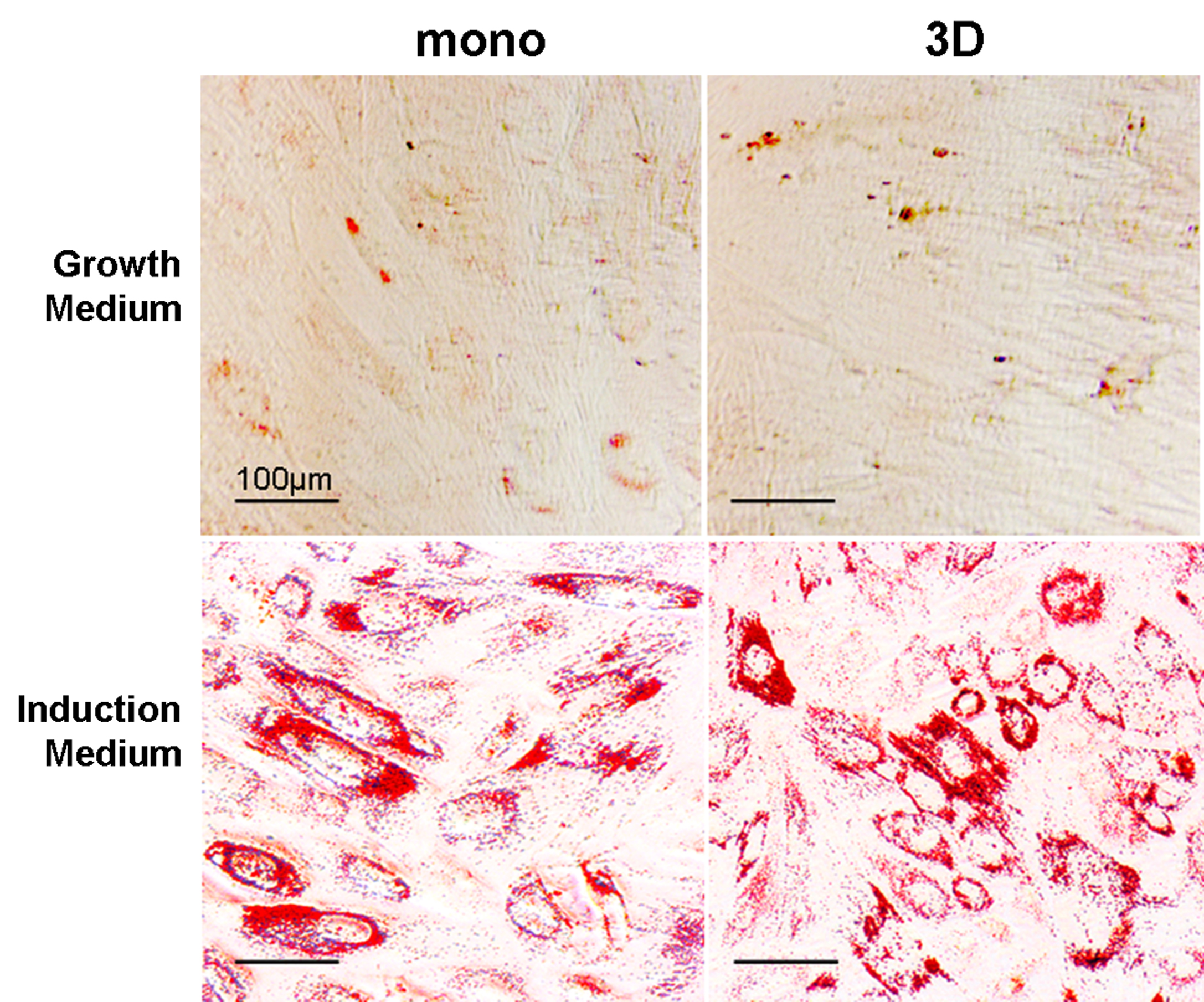

Supplement: Figure S2 — Adipogenic differentiation of hMSCs. Human MSCs derived from monolayer and spheroids (3D) were cultured in the growth medium or adipogenic induction medium for 14 days. Cells were photographed after staining with Oil Red O for detection of adipogenesis. The experiment was repeated three times with similar results and results from one experiment were shown. [file jcmm0018-2009-sd2.jpg]
