# Supplementary material for: Epigenetic changes of mesenchymal stem cells in three-dimensional (3D) spheroids
Source: J Cell Mol Med. 2014 Aug 5;18(10):2009–19. doi: 10.1111/jcmm.12336 (PMC4244016; doi:10.1111/jcmm.12336)
Supplement: Supplementary file 3 [file jcmm0018-2009-sd3.ppt]

## Slide 1
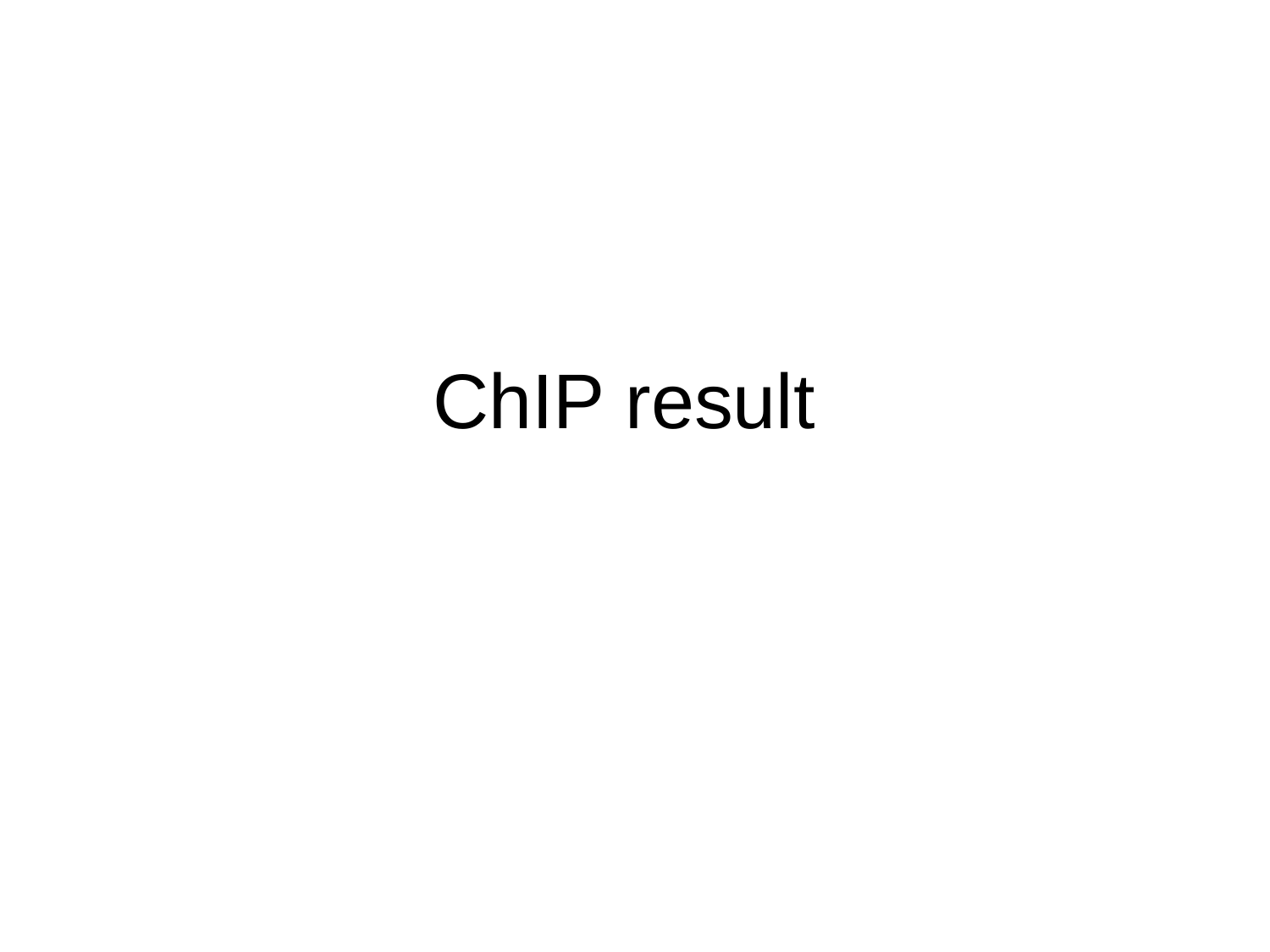

# ChIP result

## Slide 2
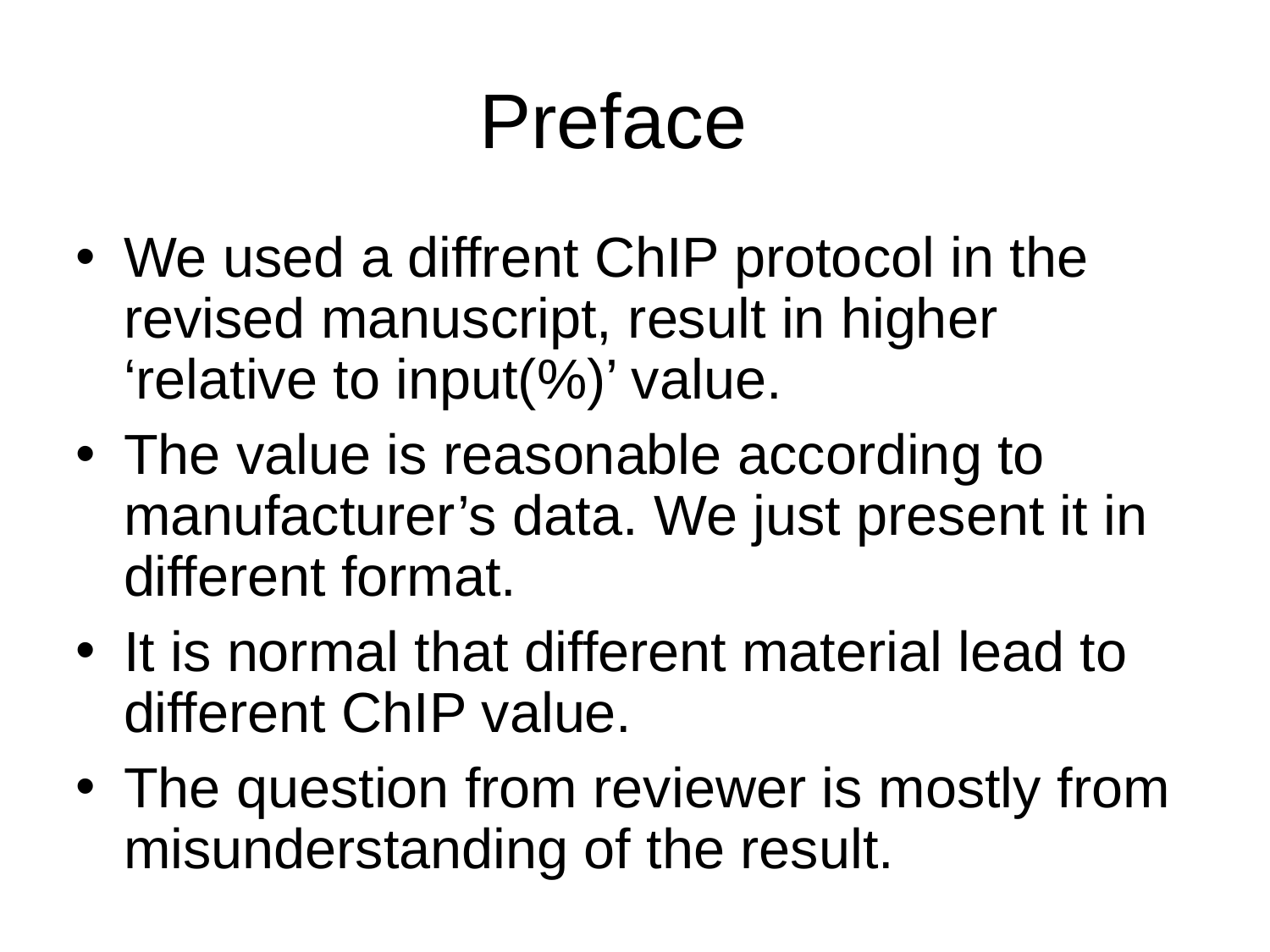

# Preface
We used a diffrent ChIP protocol in the revised manuscript, result in higher ‘relative to input(%)’ value.
The value is reasonable according to manufacturer’s data. We just present it in different format.
It is normal that different material lead to different ChIP value.
The question from reviewer is mostly from misunderstanding of the result.

## Slide 3
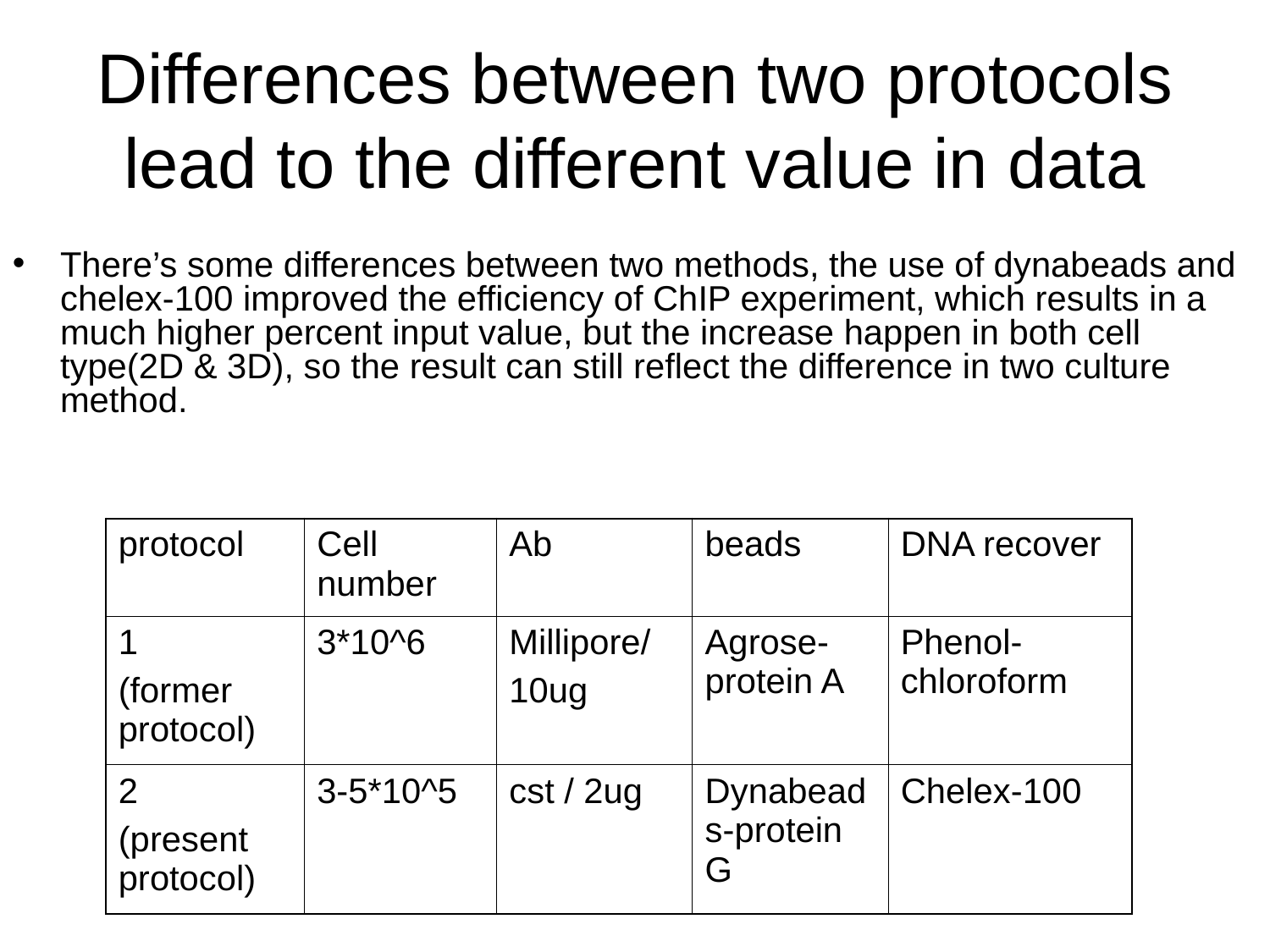

# Differences between two protocols lead to the different value in data
There’s some differences between two methods, the use of dynabeads and chelex-100 improved the efficiency of ChIP experiment, which results in a much higher percent input value, but the increase happen in both cell type(2D & 3D), so the result can still reflect the difference in two culture method.
| protocol | Cell number | Ab | beads | DNA recover |
| --- | --- | --- | --- | --- |
| 1 (former protocol) | 3\*10^6 | Millipore/ 10ug | Agrose-protein A | Phenol-chloroform |
| 2 (present protocol) | 3-5\*10^5 | cst / 2ug | Dynabeads-protein G | Chelex-100 |

## Slide 4
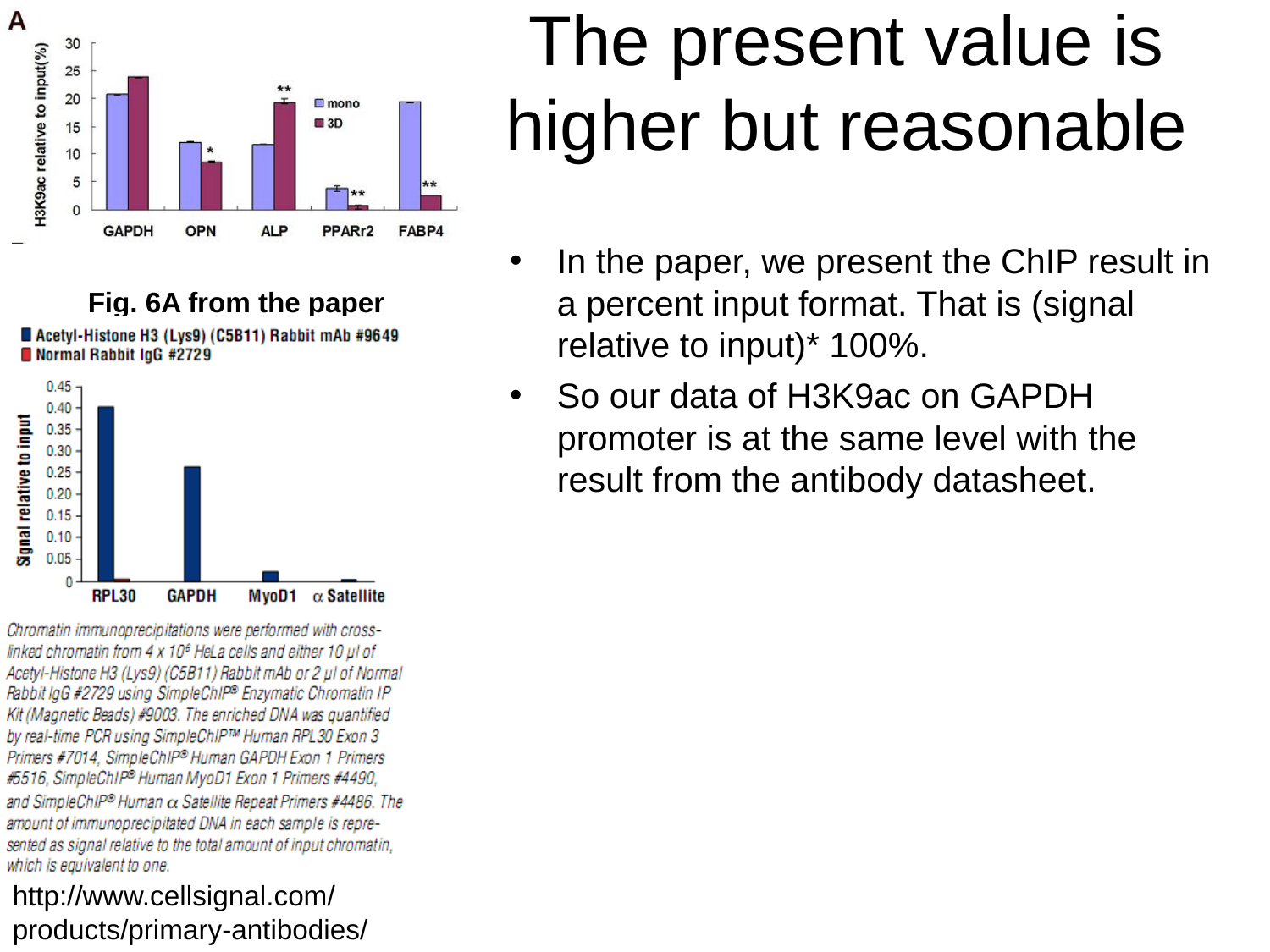

# The present value is higher but reasonable
In the paper, we present the ChIP result in a percent input format. That is (signal relative to input)* 100%.
So our data of H3K9ac on GAPDH promoter is at the same level with the result from the antibody datasheet.
Fig. 6A from the paper
http://www.cellsignal.com/products/primary-antibodies/9649

## Slide 5
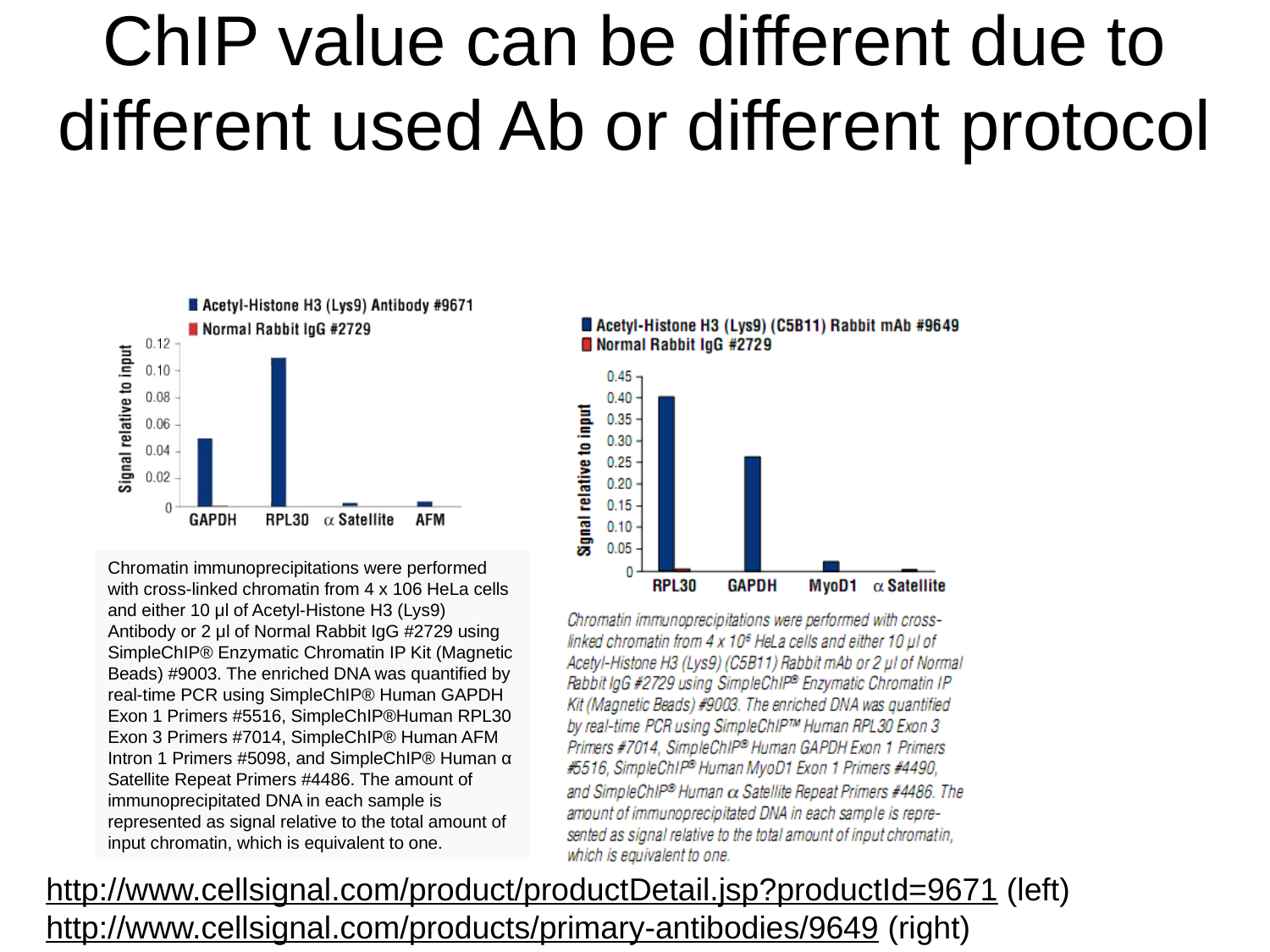

# ChIP value can be different due to different used Ab or different protocol
Chromatin immunoprecipitations were performed with cross-linked chromatin from 4 x 10^6^ HeLa cells and either 10 μl of Acetyl-Histone H3 (Lys9) Antibody or 2 μl of Normal Rabbit IgG #2729 using SimpleChIP® Enzymatic Chromatin IP Kit (Magnetic Beads) #9003. The enriched DNA was quantified by real-time PCR using SimpleChIP® Human GAPDH Exon 1 Primers #5516, SimpleChIP®Human RPL30 Exon 3 Primers #7014, SimpleChIP® Human AFM Intron 1 Primers #5098, and SimpleChIP® Human α Satellite Repeat Primers #4486. The amount of immunoprecipitated DNA in each sample is represented as signal relative to the total amount of input chromatin, which is equivalent to one.
http://www.cellsignal.com/product/productDetail.jsp?productId=9671 (left)
http://www.cellsignal.com/products/primary-antibodies/9649 (right)

## Slide 6
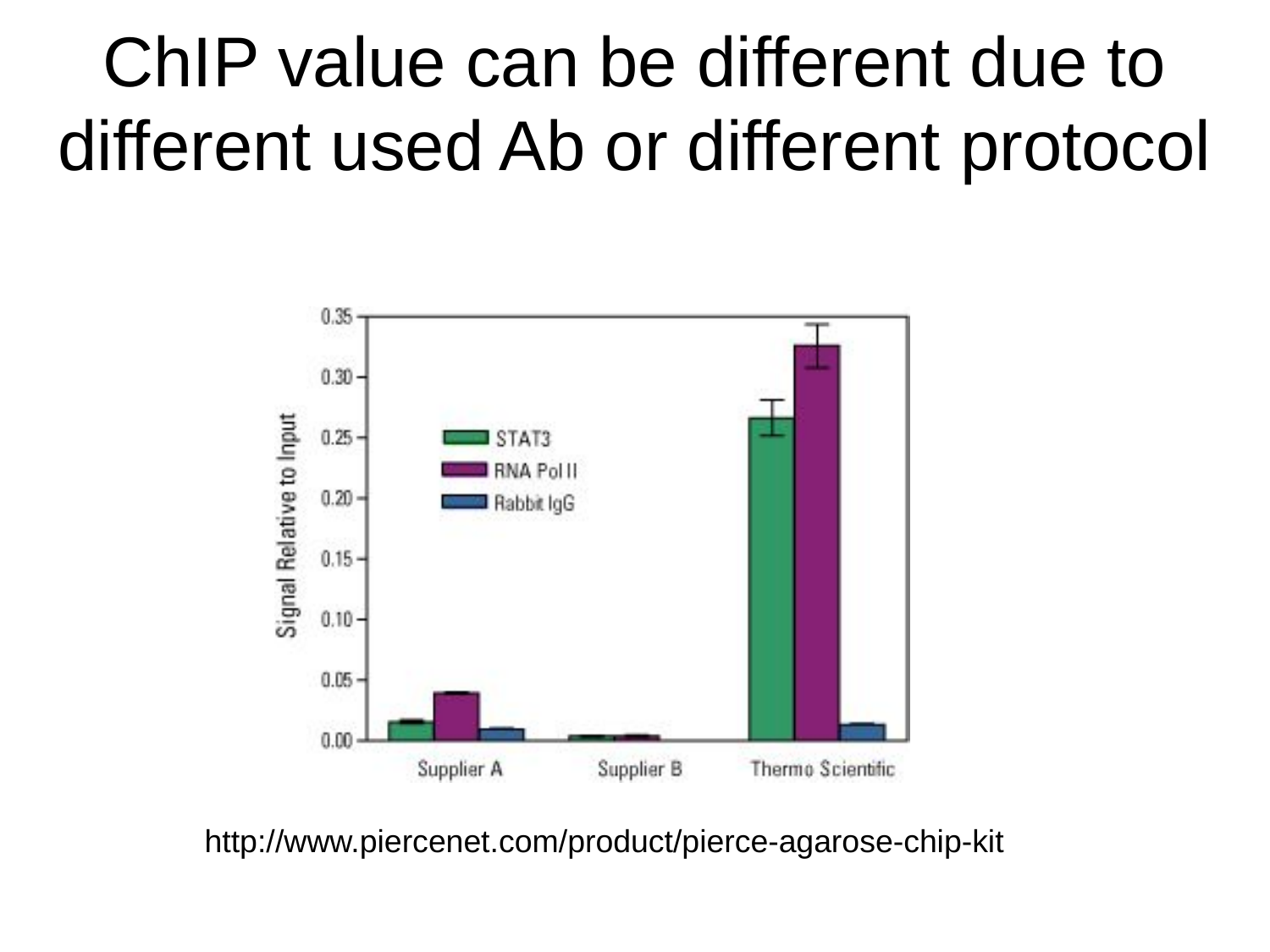

# ChIP value can be different due to different used Ab or different protocol
http://www.piercenet.com/product/pierce-agarose-chip-kit

## Slide 7
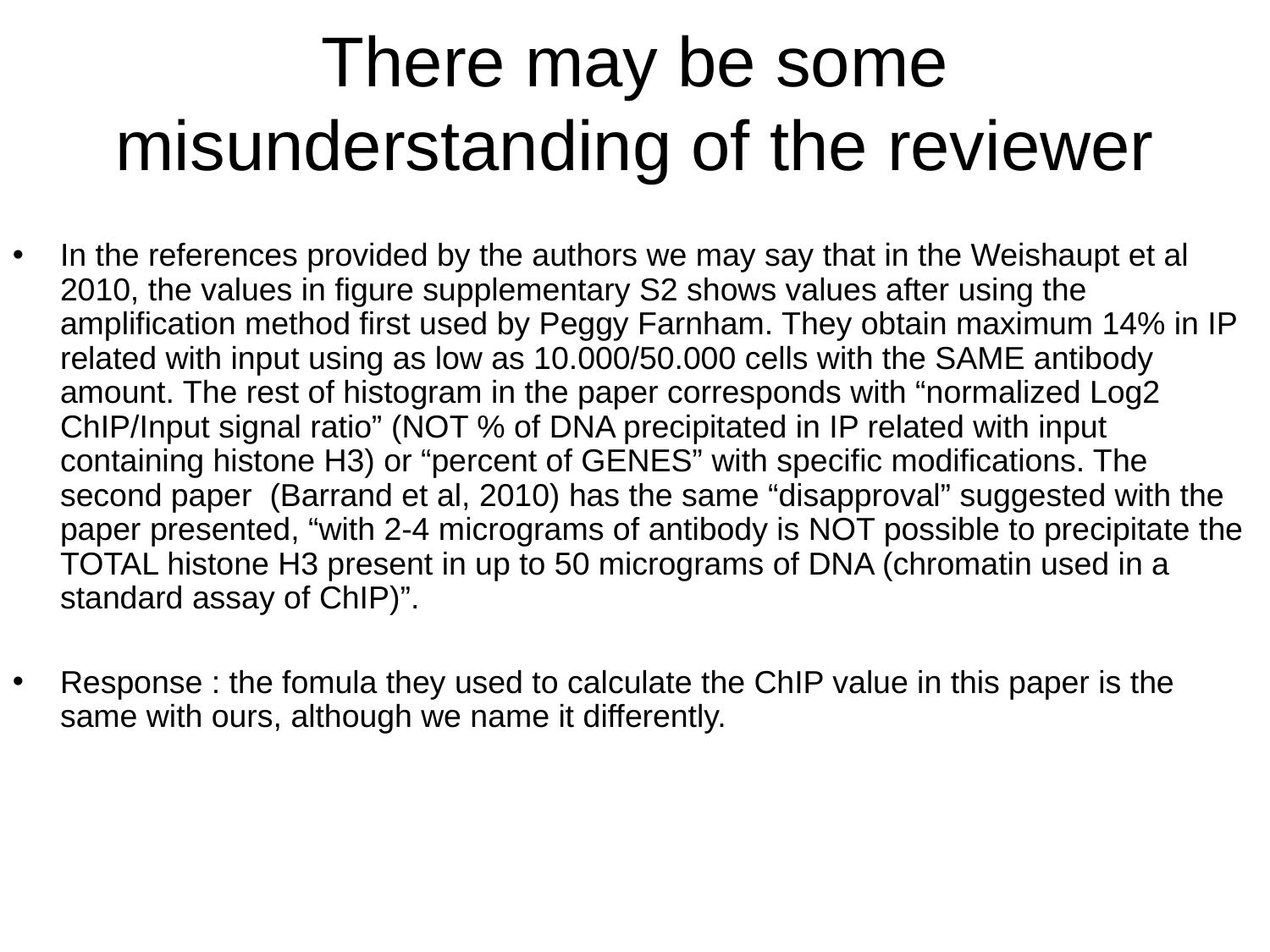

# There may be some misunderstanding of the reviewer
In the references provided by the authors we may say that in the Weishaupt et al 2010, the values in figure supplementary S2 shows values after using the amplification method first used by Peggy Farnham. They obtain maximum 14% in IP related with input using as low as 10.000/50.000 cells with the SAME antibody amount. The rest of histogram in the paper corresponds with “normalized Log2 ChIP/Input signal ratio” (NOT % of DNA precipitated in IP related with input containing histone H3) or “percent of GENES” with specific modifications. The second paper (Barrand et al, 2010) has the same “disapproval” suggested with the paper presented, “with 2-4 micrograms of antibody is NOT possible to precipitate the TOTAL histone H3 present in up to 50 micrograms of DNA (chromatin used in a standard assay of ChIP)”.
Response : the fomula they used to calculate the ChIP value in this paper is the same with ours, although we name it differently.

## Slide 8
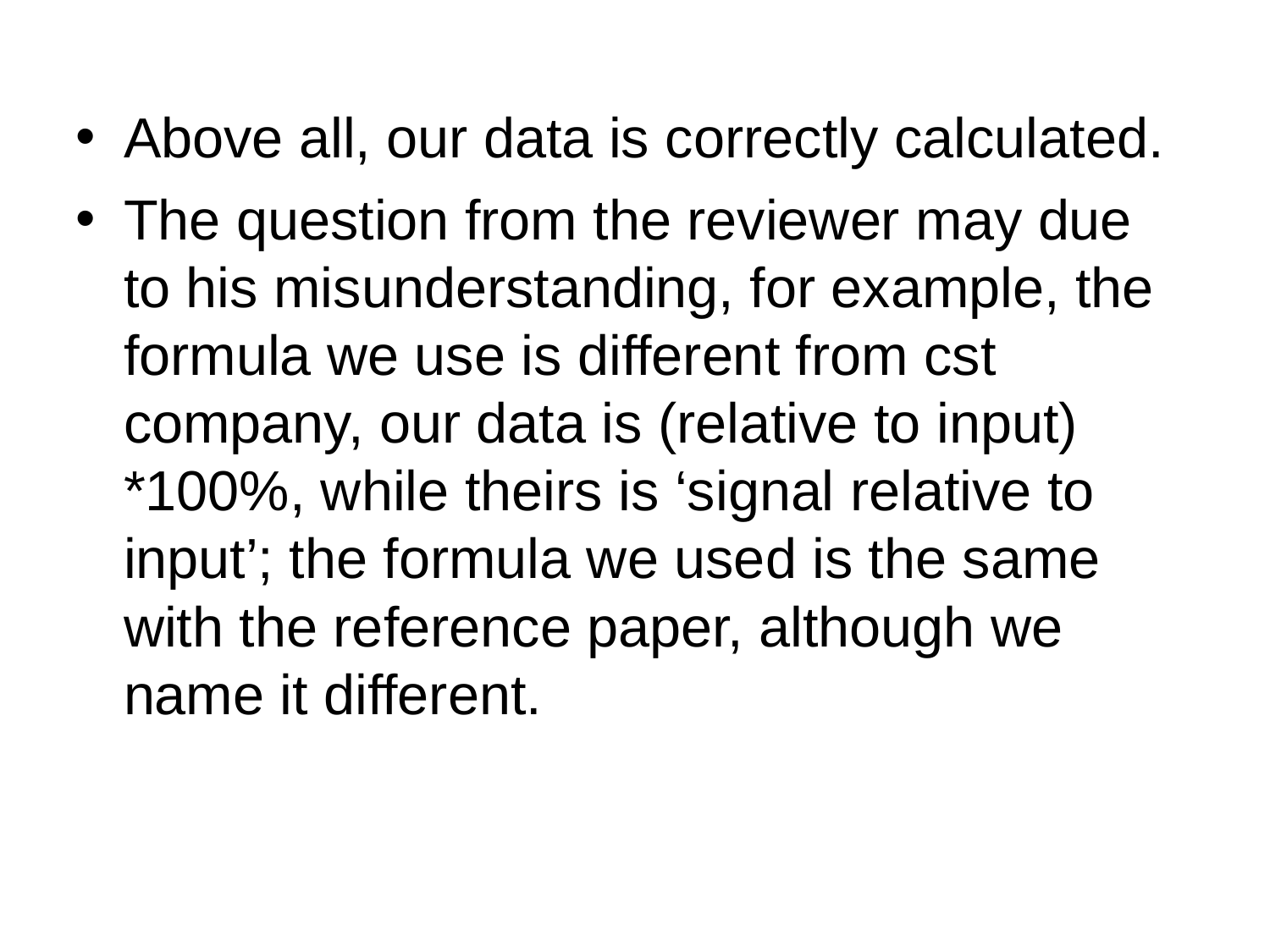

# Above all, our data is correctly calculated.
The question from the reviewer may due to his misunderstanding, for example, the formula we use is different from cst company, our data is (relative to input) *100%, while theirs is ‘signal relative to input’; the formula we used is the same with the reference paper, although we name it different.
